# Preoperative albumin-to-globulin ratio as a prognostic factor in patients undergoing curative hepatectomy for hepatocellular carcinoma: A systematic review and meta-analysis

**DOI:** 10.1097/MD.0000000000049830

**Published:** 2026-07-17

**Authors:** Masashi Utsumi, Masaru Inagaki, Koki Omoto, Naoki Onoda, Koji Kitada, Naoyuki Tokunaga, Hiroki Okabayashi, Ryosuke Hamano, Hideaki Miyaso, Yosuke Tsunemitsu, Shinya Otsuka

**Affiliations:** a Department of Surgery, NHO Fukuyama Medical Center, Fukuyama City, Hiroshima, Japan.

**Keywords:** albumin-to-globulin ratio, hepatocellular carcinoma, meta-analysis, prognosis

## Abstract

**Background:**

The albumin-to-globulin ratio (AGR) has emerged as a novel inflammation-based prognostic marker in various cancers; however, a comprehensive and quantitative assessment of its prognostic role in hepatocellular carcinoma (HCC) is lacking. Therefore, we conducted this meta-analysis to assess the prognostic value of the preoperative AGR in patients with HCC.

**Methods:**

We systematically reviewed the literature for studies reporting the prognostic impact of AGR in patients undergoing curative hepatectomy for HCC. Random-effects meta-analyses of overall survival (OS) and recurrence-free survival were performed.

**Results:**

Six studies involving 2107 patients were included in this meta-analysis, with all conducted in Asian populations. Four studies were conducted in China, whereas 2 studies were conducted in Japan. Low pretreatment AGR was significantly correlated with decreased OS (hazard ratio: 1.69, 95% confidence interval: 1.37–2.08, *P* < .001) and recurrence-free survival (hazard ratio: 1.62, 95% confidence interval: 1.37–1.91, *P* < .001). Subgroup analyses suggested that a low AGR predicted decreased OS in patients with HCC, regardless of region, sample size, age, and cutoff values.

**Conclusion:**

Low preoperative AGR was significantly associated with poor prognosis of patients with HCC. AGR may serve as a prognostic biomarker, although validation in prospective cohorts is required.

## 1. Introduction

Hepatocellular carcinoma (HCC) remains one of the most common malignancies worldwide and continues to rise in incidence, ranking among the leading causes of cancer-related mortality.^[1]^ Surgical resection offers the best chance of long-term survival for patients with adequate hepatic reserve.^[2]^ However, most patients fail to undergo curative surgery because HCC often presents with features such as vascular invasion, extrahepatic spread, or an asymptomatic early course that delays diagnosis.^[3]^ Despite advances in imaging, perioperative management, and surgical techniques, overall outcomes remain unsatisfactory, underscoring the need for reliable prognostic indicators that can be assessed before treatment.

Increasing evidence highlights the critical role of systemic inflammation in cancer development and progression.^[4]^ Simple blood-based inflammatory markers – including the platelet-to-lymphocyte ratio and neutrophil-to-lymphocyte ratio – have been widely investigated as prognostic tools across multiple tumor types.^[5,6]^ These indices are attractive because they are inexpensive, routinely measured, and easily obtainable from standard preoperative laboratory tests. Serum protein components also provide clinically relevant information: albumin reflects nutritional status and hepatic synthetic function, whereas globulin levels rise in response to chronic inflammation and immune activation.^[7]^

The albumin-to-globulin ratio (AGR), which integrates these 2 parameters, has been reported as a prognostic marker in several malignancies, including gastric, colorectal, and breast cancers.^[8–10]^ However, its prognostic significance in patients undergoing curative hepatectomy for HCC has not been comprehensively evaluated. To address this gap, we performed a systematic review and meta-analysis to clarify the association between preoperative AGR and postoperative outcomes in patients with HCC.

## 2. Materials and methods

### 2.1. Guidelines and ethics

This systematic review and meta-analysis was conducted in accordance with the Preferred Reporting Items for Systematic Reviews and Meta-Analyses guidelines.^[11]^ Because all data were obtained from previously published studies, no ethical approval or informed consent was required.

### 2.2. Search strategy

A comprehensive literature search was carried out using PubMed, Cochrane Central, ProQuest, and Google Scholar. The final search update was performed on March 1, 2025. To improve reproducibility when using Google Scholar, the first 200 search results (approximately 20 pages) were screened. The search strategy combined terms related to the AGR (“albumin-to-globulin ratio,” “albumin to globulin,” “albumin globulin ratio,” “albumin/globulin,” “albumin and globulin,” “AGR”) with terms related to HCC (“hepatocellular carcinoma,” “hepatocellular cancer,” “HCC”). Only English-language publications were considered. Reference lists of eligible articles, relevant reviews, and previous meta-analyses were also manually searched to identify additional studies.

### 2.3. Eligibility criteria

Studies were included if they met all of the following criteria:

1. HCC diagnosis confirmed by pathological examination;
2. preoperative AGR measured using blood tests prior to hepatectomy;
3. reported associations between AGR and overall survival (OS) and/or recurrence-free survival (RFS); and
4. provided sufficient data to extract or calculate hazard ratios (HRs) with 95% confidence intervals (CI).

Exclusion criteria were as follows:

1. reviews, letters, case reports, or conference abstracts lacking original data;
2. studies without adequate information for quantitative analysis;
3. studies including patients who did not undergo hepatectomy;
4. duplicate or overlapping datasets; and
5. non-English publications.

### 2.4. Data extraction and quality assessment

Two reviewers independently screened eligible studies and extracted data. Discrepancies were resolved through discussion among all authors. Extracted variables included first author, publication year, study design, country, recruitment period, sample size, patient demographics, AGR cutoff values and determination methods, treatment details, survival outcomes, HRs with 95% CIs for OS and RFS, and clinicopathological characteristics. When both univariable and multivariable HRs were available, multivariable estimates were prioritized; univariable HRs were used only when multivariable data were not reported.

Study quality was assessed using the Newcastle–Ottawa Scale,^[12]^ which evaluates selection, comparability, and outcome domains, with scores ranging from 0 to 9 (Table S1, Supplemental Digital Content 1). Studies scoring ≥6 were considered high quality. In addition to Newcastle–Ottawa Scale scoring, we qualitatively evaluated the certainty of evidence using GRADE domains, including risk of bias, inconsistency, indirectness, imprecision, and publication bias.

### 2.5. Statistical analysis

Meta-analysis was performed using Review Manager (RevMan), version 5.4 (Cochrane Collaboration, 2020). Pooled risk ratios with 95% CIs were calculated for dichotomous outcomes using random-effects models. When heterogeneity was present, subgroup analyses were conducted to explore potential sources. Statistical heterogeneity was assessed using the chi-square test and the *I*^2^ statistic, with *P* <.05 indicating significance and *I*^2^ ≥50% suggesting substantial heterogeneity. Funnel plots were generated to evaluate potential publication bias.

## 3. Results

### 3.1. Retrieval of literature and study characteristics

**Figure 1.**
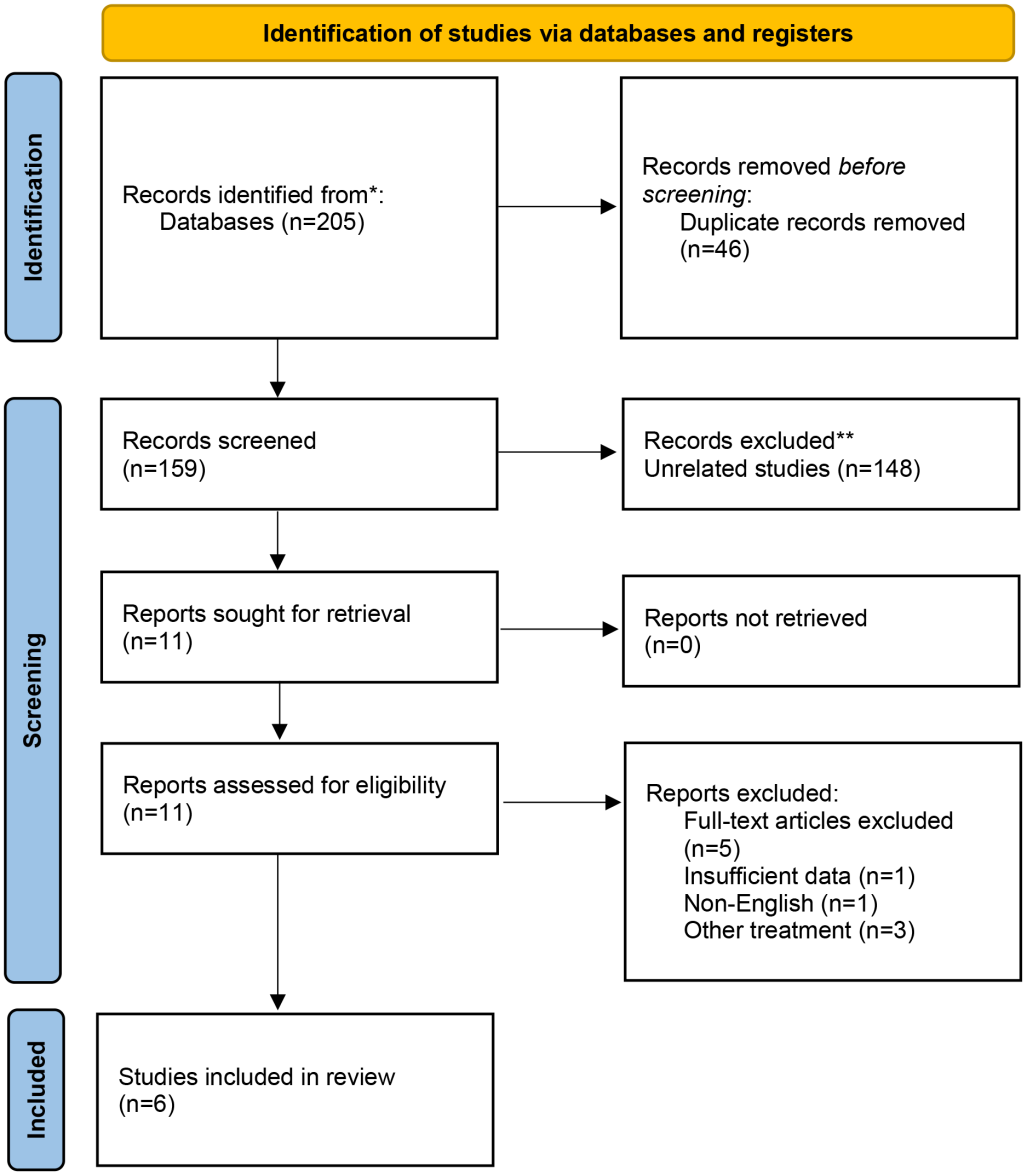
Flow diagram of study selection. SE = standard error.

The study selection process is summarized in Figure 1. A total of 205 records were initially identified across all databases, and 46 duplicates were removed. After screening titles and abstracts, 159 articles remained, of which 148 were excluded for not meeting the eligibility criteria. Eleven full-text articles were reviewed in detail, and 5 articles were excluded: 1 article lacked extractable data, 1 article was not published in English, and 3 articles involved patients with HCC who did not undergo surgical resection. Ultimately, 6 studies comprising 2107 patients who received curative hepatectomy were included in the final analysis. The baseline characteristics of these studies are presented in Table 1.^[13–18]^

**Table 1:** Characteristics of the studies included in the meta-analysis.

Author	Yr	Country	Sample size	Age, median or mean (yr)	Study design	Stage	Cutoff value	Survival data	NOS score
Deng^[13]^	2016	China	172	53	Retrospective	Mixed	1.48	OS/RFS	7
Shimizu^[14]^	2017	Japan	368	≥65	Retrospective	Mixed	1.09	OS	7
Zhang^[15]^	2019	China	210	55	Retrospective	Mixed	1.4	OS	6
Zhang^[16]^	2020	China	693	51.5	Retrospective	Mixed	1.00	OS/RFS	7
Utsumi^[17]^	2021	Japan	157	73	Retrospective	Mixed	1.16	OS/RFS	6
Li^[18]^	2025	China	507	54	Retrospective	Mixed	1.79	OS/RFS	7

NOS = Newcastle–Ottawa Scale, OS = overall survival, RFS = recurrence-free survival.

One study reported the globulin-to-albumin ratio rather than AGR; this value was converted to AGR after repeated verification among the authors. Sample sizes ranged from 157 to 693 patients. All included studies were retrospective and published between 2016 and 2025. Four studies originated from China,^[13,15,16,18]^ while 2 studies were conducted in Japan.^[14,17]^ All studies achieved an NOS score of ≥6, indicating acceptable methodological quality (Table S1, Supplemental Digital Content 1). Based on a qualitative GRADE assessment, the overall certainty of evidence was judged to be low, primarily due to the retrospective nature of all included studies, the modest number of available datasets, and the possibility of publication bias, despite relatively low statistical heterogeneity.

### 3.2. AGR and OS

**Figure 2.**
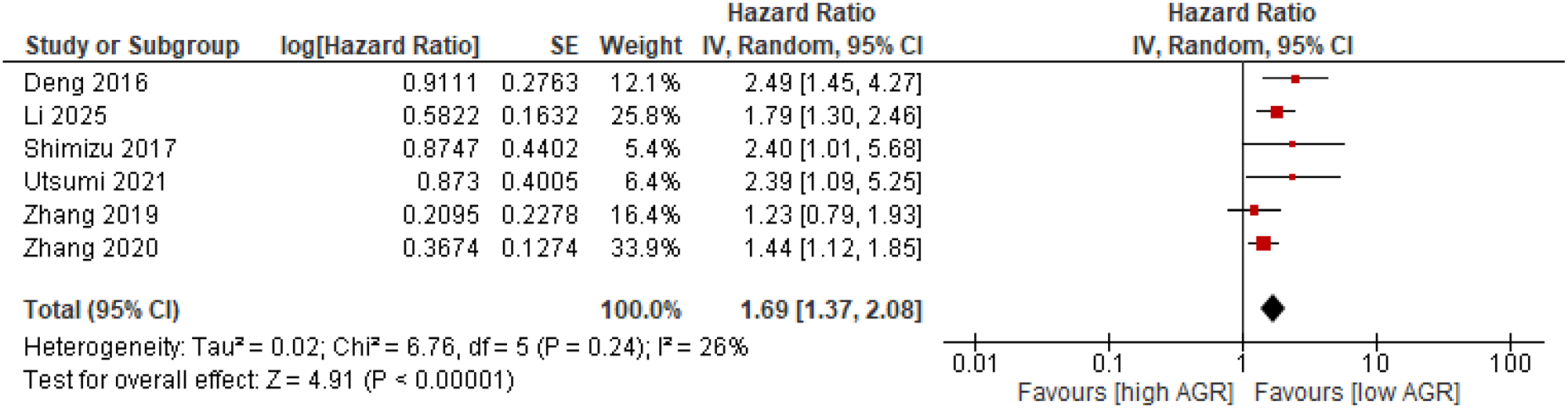
Forest plot of the association between AGR and overall survival. AGR = albumin-to-globulin ratio, CI = confidence interval, IV = inverse variance, SE = standard error.

All 6 studies (n = 2107) reported the association between AGR and OS. Patients with low AGR consistently demonstrated poorer OS, with a pooled HR of 1.69 (95% CI: 1.37–2.08, *P* < .001) using a random-effects model. Heterogeneity was low to moderate (*I*^2^ = 26%, *P* = .24; Fig. 2). Subgroup analyses stratified by sample size, geographic region, patient age, and AGR cutoff values showed uniformly significant associations (all *P* < .05), supporting the robustness of AGR as a prognostic indicator for OS in HCC (Table 2).

**Table 2:** Subgroup analysis of the correlation between AGR and OS in different factors.

Subgroup factor	Number of studies	Number of patients	HR (95% CI)	*P* value	Heterogeneity	
					*I*^2^ (%)	*P*_h_
OS	6	2107	1.69 (1.37–2.08)	<.001	26	.24
Region						
China	4	1582	1.61 (1.33–2.03)	<.001	40	.17
Japan	2	525	2.40 (1.34–4.28)	.003	0	1.00
Sample size						
<300	3	539	1.85 (1.12–3.06)	.020	56	.10
≥300	3	1568	1.60 (1.32–1.94)	<.001	0	.37
Age (median or mean)						
<60	4	1582	1.61 (1.33–2.03)	<.001	40	.17
≥60	2	525	2.40 (1.34–4.98)	.003	0	1.00
AGR cutoff value						
<1.10	2	1061	1.56 (1.09–2.24)	.020	18	.27
≥1.10	4	1046	1.81 (1.41–2.32)	<.001	17	.30

AGR = albumin-to-globulin ratio, CI = confidence interval, HR = hazard ratio, OS = overall survival, RFS = recurrence-free survival.

### 3.3. AGR and RFS

**Figure 3.**
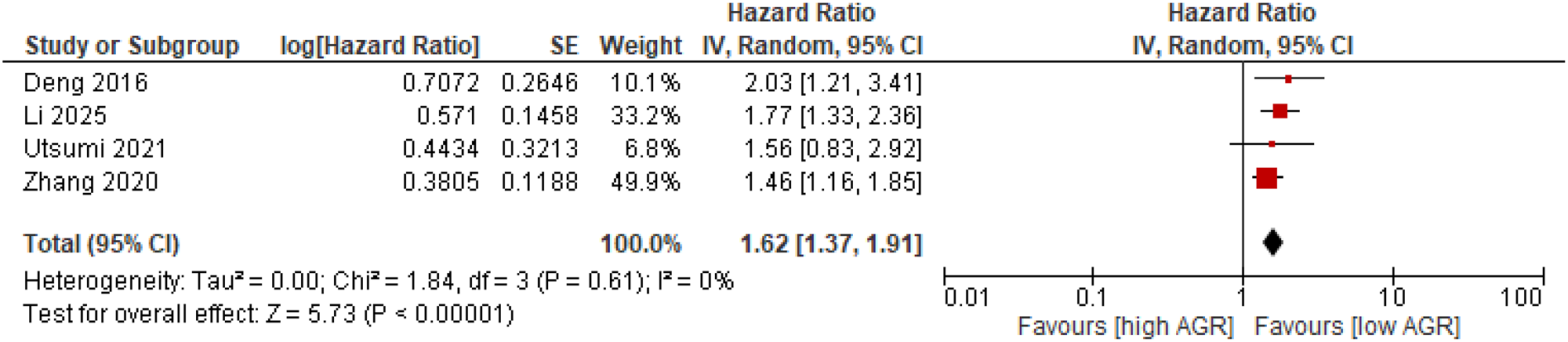
Forest plot of the association between AGR and recurrence-free survival. AGR = albumin-to-globulin ratio, CI = confidence interval, IV = inverse variance, SE = standard error.

Four studies involving 1529 patients provided data on RFS. Low AGR was associated with significantly shorter RFS (HR: 1.62, 95% CI: 1.37–1.91, *P* < .001). No heterogeneity was observed (*I*^2^ = 0%, *P* = .61; Fig. 3), indicating a highly consistent effect across studies.

### 3.4. AGR and clinicopathological factors

Associations between AGR and clinicopathological characteristics are summarized in Table 3. Compared with patients with high AGR, those with low AGR were less likely to have Child–Pugh grade A liver function (pooled odds ratio: 4.67 favoring the high-AGR group). Conversely, liver cirrhosis was more common among patients with low AGR (pooled odds ratio: 0.57). No significant associations were identified for hepatitis B virus infection, tumor multiplicity, or microvascular invasion. These findings suggest that low AGR is linked to poorer hepatic reserve and more advanced underlying liver disease.

**Table 3:** Associations between low AGR (vs high AGR) and clinicopathological characteristics.

Variable	Number of studies	OR (95% CI)	*P* value	*I*^2^ (%)	Analysis model
Child–Pugh A	3	4.67 (2.84–7.67)	<.001	0	Random
Liver cirrhosis	4	0.57 (0.44–0.75)	<.001	0	Random
HBV infection	4	1.42 (0.87–2.30)	.16	47	Random
Multiply tumors	4	0.79 (0.57–1.09)	.15	13	Random
Microvascular invasion	4	0.63 (0.34–1.17)	.14	76	Random

AGR = albumin-to-globulin ratio, CI = confidence interval, HBV = hepatitis B virus, OR = odds ratio.

### 3.5. Publication bias

Visual inspection of funnel plots did not reveal substantial asymmetry, suggesting no clear evidence of publication bias (Fig. S1, Supplemental Digital Content 2). However, the small number of included studies limits the reliability of this assessment.

## 4. Discussion

This meta-analysis synthesized data from 6 retrospective studies, including 2107 patients who underwent curative hepatectomy for HCC, and demonstrated a consistent association between low preoperative AGR and poorer postoperative outcomes. Both OS and RFS were significantly worse in patients with reduced AGR, suggesting that this simple biochemical index may capture important aspects of host physiology relevant to prognosis. The observation that low AGR was also linked to impaired liver function further supports the notion that AGR reflects a combination of nutritional status, hepatic reserve, and systemic inflammation.

The biological rationale for the prognostic value of AGR is grounded in the well-established relationship between chronic inflammation and carcinogenesis. Tumors frequently arise in inflammatory environments, and inflammatory cells are commonly detected within tumor tissues.^[19]^ Experimental and clinical studies have shown that inflammation promotes tumor growth, invasion, and metastasis, and the inflammatory microenvironment has been described as a hallmark of cancer.^[20–22]^ Consequently, inflammation-based biomarkers have been widely investigated as prognostic tools across malignancies, including HCC.

HCC prognosis is influenced not only by tumor burden but also by underlying liver function.^[23]^ Several inflammation- and nutrition-related indices – such as neutrophil-to-lymphocyte ratio, platelet-to-lymphocyte ratio, prognostic nutritional index, and controlling nutrition – have been associated with survival in HCC.^[24–28]^ However, each of these markers captures only part of the complex interplay between inflammation, nutrition, and hepatic function. Albumin reflects hepatic synthetic capacity and nutritional reserve, whereas globulin levels rise in response to immune activation and chronic inflammation.^[7]^ AGR integrates these 2 components, making it a potentially more comprehensive indicator of the host condition.

AGR is calculated by dividing serum albumin by the difference between total protein and albumin, the latter representing the globulin fraction. Elevated globulin levels reflect increased production of inflammatory proteins.^[29]^ Systemic inflammation contributes to tumor progression by altering cellular signaling, promoting proliferation, and suppressing antitumor immunity.^[30]^ Cytokines such as interleukin-1, interleukin-6, and tumor necrosis factor stimulate the hepatic synthesis of acute-phase proteins,^[31–33]^ many of which are contained within the globulin fraction, including C-reactive protein, serum amyloid A, and fibrinogen.^[34]^ In HCC, the peritumoral stroma can further amplify inflammatory pathways through innate immune activation, thereby facilitating tumor progression.^[35,36]^

In contrast, albumin plays multiple physiological roles beyond maintaining oncotic pressure. Hypoalbuminemia has been associated with poor outcomes in various cancers,^[37–39]^ and in HCC, it may reflect both malnutrition and impaired hepatic synthetic function.^[40]^ It acts as an antioxidant, stabilizes cellular processes, and participates in hormone and drug transport.^[41]^ Reduced albumin levels can weaken immune defenses, increase susceptibility to infection, and exacerbate cytokine-mediated catabolism, all of which may contribute to inferior long-term survival.^[42–44]^ Taken together, a low AGR likely represents the combined effects of heightened inflammation, diminished hepatic reserve, and nutritional decline.

Given these considerations, AGR may serve as a surrogate marker for the overall physiological resilience of patients undergoing hepatectomy. Nutritional impairment is common among patients with hepatobiliary malignancies, and perioperative nutritional support has been recommended to improve outcomes.^[45]^ Immunonutrition has also been shown to attenuate postoperative inflammatory responses.^[46]^ Future studies should explore whether interventions targeting nutrition or inflammation can modify AGR and potentially improve postoperative outcomes.

This study has several limitations. First, the number of included studies and total sample size were limited, reducing statistical power. Second, AGR was assessed only at a single preoperative time point, and its dynamic changes during treatment were not evaluated. Third, AGR cutoff values varied across studies, which may have contributed to heterogeneity. Fourth, all included studies were retrospective and conducted in Asian populations, limiting generalizability to other regions. In addition, some studies reported only univariable HRs, whereas others provided multivariable estimates, raising the possibility of residual confounding. Publication bias cannot be excluded, as most included studies reported positive findings. Although we qualitatively assessed certainty using GRADE principles, no formal evidence profile was constructed, which may limit transparency. Larger studies, including those with negative results, are needed to validate these findings.

From a clinical perspective, patients with low AGR may be at higher risk of poor postoperative outcomes. However, AGR should not be used in isolation to guide treatment decisions. Established prognostic factors – including liver function (Child–Pugh or albumin–bilirubin score), tumor burden, portal hypertension, alpha-fetoprotein levels, performance status, and Barcelona clinic liver cancer stage – must remain central to treatment selection. The potential role of AGR in decision-making should be evaluated in prospective studies and incorporated into multivariable prognostic models before clinical adoption.

In summary, low preoperative AGR was associated with significantly reduced OS and RFS in patients undergoing curative hepatectomy for HCC. Although AGR is an inexpensive and easily obtainable biomarker, its clinical utility requires confirmation in well-designed prospective studies.

## 5. Conclusion

This meta-analysis demonstrated that a reduced preoperative AGR is consistently associated with poorer overall and RFS in patients undergoing curative hepatectomy for HCC. As a readily available and inexpensive laboratory parameter, AGR has the potential to complement existing prognostic tools by reflecting the combined influence of nutritional status, hepatic functional reserve, and systemic inflammation. However, given the retrospective nature of the available evidence and the limited number of studies, the clinical applicability of AGR remains uncertain. Well-designed prospective investigations are needed to validate its prognostic value and to determine whether AGR can be effectively incorporated into future risk-stratification models for HCC.

## Acknowledgments

We would like to thank Editage (www.editage.com) for the English language editing.

## Author contributions

**Conceptualization:** Ryosuke Hamano, Yosuke Tsunemitsu, Shinya Otsuka.

**Data curation:** Masashi Utsumi, Koji Kitada, Hiroki Okabayashi.

**Investigation:** Masashi Utsumi, Koki Omoto, Naoki Onoda.

**Project administration:** Masashi Utsumi.

**Supervision:** Masaru Inagaki, Naoyuki Tokunaga, Hideaki Miyaso, Shinya Otsuka.

**Writing – original draft:** Masashi Utsumi.

**Writing – review & editing:** Masaru Inagaki, Shinya Otsuka.




