# Supplementary material for: Preoperative albumin-to-globulin ratio as a prognostic factor in patients undergoing curative hepatectomy for hepatocellular carcinoma: A systematic review and meta-analysis
Source: Medicine (Baltimore). 2026 Jul 17;105(29):e49830. doi: 10.1097/MD.0000000000049830 (PMC13384584; doi:10.1097/MD.0000000000049830)
Supplement: Supplementary file 1 [file medi-105-e49830-s001.docx]

Table S1. Newcastle-Ottawa Scale used for assessing quality of studies in meta-analysis

| Studies | Selection | | | | Comparability | Exposure | | | Total Quality score |
| --- | --- | --- | --- | --- | --- | --- | --- | --- | --- |
| Author, year | Is the Case Definition Adequate? | Representativeness of the Cases | Selection of Controls | Definition of Controls | Comparability of cases and controls | Ascertainment of exposure | Same method of ascertainment for cases and controls | Non-Response rate |  |
| Deng (18), 2016 | 1 | 1 | 0 | 1 | 1 | 1 | 1 | 1 | 7 |
| Shimizu (13), 2017 | 0 | 1 | 1 | 1 | 1 | 1 | 1 | 1 | 7 |
| Zhang (14), 2019 | 1 | 1 | 0 | 1 | 1 | 1 | 1 | 0 | 6 |
| Zhang (15), 2020 | 1 | 1 | 0 | 1 | 1 | 1 | 1 | 1 | 7 |
| Utsumi (16), 2021 | 1 | 1 | 0 | 1 | 1 | 1 | 1 | 0 | 6 |
| Li (17), 2025 | 1 | 1 | 0 | 1 | 1 | 1 | 1 | 1 | 7 |
