# Supplementary figures and images for: Preoperative albumin-to-globulin ratio as a prognostic factor in patients undergoing curative hepatectomy for hepatocellular carcinoma: A systematic review and meta-analysis
Source: Medicine (Baltimore). 2026 Jul 17;105(29):e49830. doi: 10.1097/MD.0000000000049830 (PMC13384584; doi:10.1097/MD.0000000000049830)

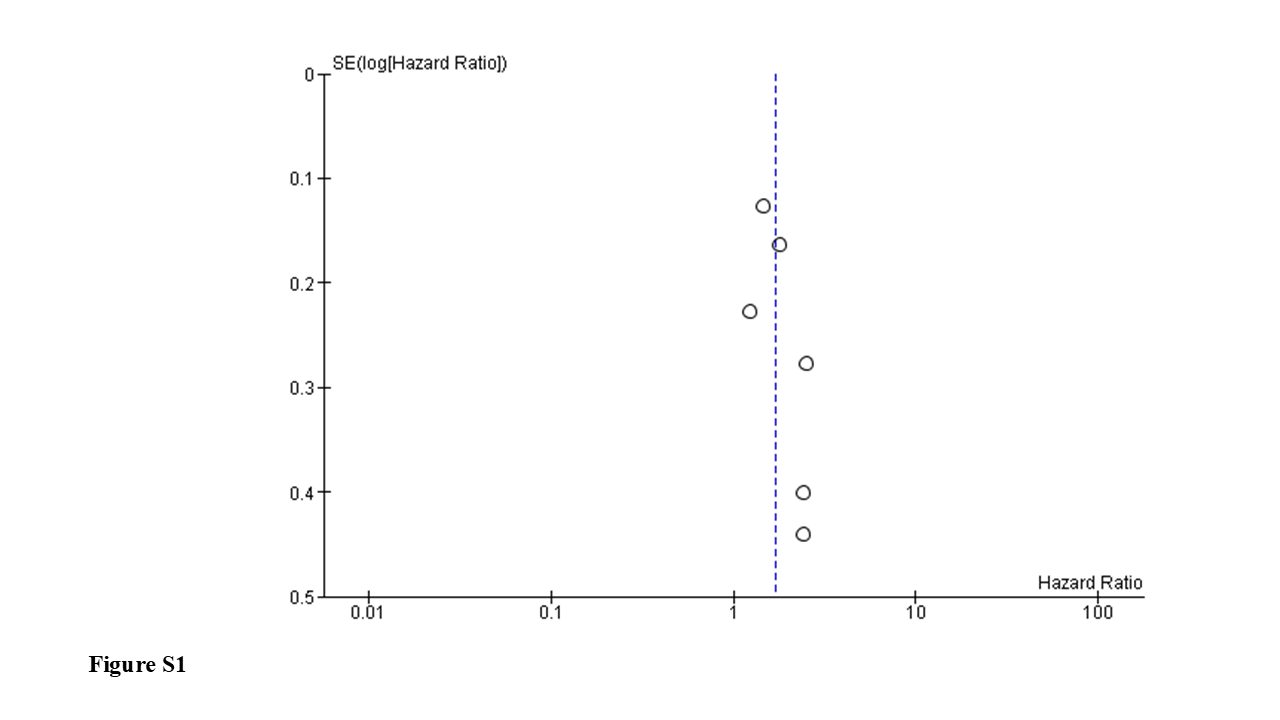

Supplement: Supplementary file 2 [file medi-105-e49830-s002.tif]
